# Analysis of Air and Soil Quality around Thermal Power Plants and Coal Mines of Singrauli Region, India

**DOI:** 10.3390/ijerph191811560

**Published:** 2022-09-14

**Authors:** Harsimranjit Kaur Romana, Ramesh P. Singh, Chandra S. Dubey, Dericks P. Shukla

**Affiliations:** 1 School of Civil and Environmental Engineering, IIT Mandi, Mandi 175005, India; 2 School of Life and Environmental Sciences, Schmid College of Science and Technology, Chapman University, Orange, CA 92866, USA; 3 K R Mangalam University, Gurgaon 122103, India

**Keywords:** Singrauli region, Thermal Power Plants (TPPs), coal mines, air and soil quality, time series forecast analysis, geo-accumulation index

## Abstract

Singrauli region is known as the energy capital of India, as it generates nearly 21 GW of electricity, supplied to various parts of the northern India. Many coal-based Thermal Power Plants (TPPs) using coal from several nearby coal mines, and numerous industries are set up in this region which has made it as one of the highly polluted regions of India. In the present study, detailed temporal analysis and forecast of carbon dioxide (CO_2_), nitrogen dioxide (NO_2_), sulfur dioxide (SO_2_), and methane (CH_4_) concentrations retrieved from satellite data have been carried out for the periods 2005–2020. Based on the classical multiplicative model and using linear regression, the maximum concentration of CO_2_, NO_2_, SO_2_, and CH_4_ in the year 2025 is found to be 422.59 ppm, 29.28 ppm, 0.23 DU, and 1901.35 ppbv, respectively. Detailed analysis shows that carbon dioxide has a 95% correlation with all other trace gases. We have also carried out the geo-accumulation index for the presence of various contaminants in the soil of this region. The geo-accumulation index shows that soil in and around thermal power plants and coal mines is contaminated by heavy metals. The cumulative index shows that soil around Hindalco industries, Bina coal mines, Khadia coal mines, and coal-based TPPs (Anpara and Vindhayachal) are highly polluted and a threat to human population living in the region.

## 1. Introduction

The growing anthropogenic activities are the main sources associated with the changes in land, atmosphere, biosphere, and cryosphere that have direct/indirect threats to our ecosystem, on a local, regional, and global scale. Before the industrial revolution, pollution was associated with natural causes, however, after the industrial revolution in the 19th century, increasing atmospheric pollution is impacting the ecosystem. Monsoon, drought, expansion of the desert, change in the genetics of forests, and melting of snow/glaciers are a few of the consequences of increasing pollution [1,2,3,4,5,6]. The growing population pressure, rapid industrialization, and development of megacities have made developing countries more vulnerable compared to developed nations. This has deteriorated the air, water, and soil quality, in addition to land degradation, thus impacting the surrounding environment, and causing various respiratory, gastro-intestinal and cardiovascular diseases [7,8,9]. According to WHO 2016, about a million people are killed every year in India due to air pollution, which is considered as one of the high health risks in the country [10] Likewise, indirect intake of heavy metals from soil contamination can cause liver, lung, neurological, and gastrointestinal diseases [11,12,13,14].

India’s energy consumption has increased more than twice since 2000 and its energy demand is projected to increase 1.5 times by 2030. As a result, electricity generation has already increased by 84% in 2021 as compared to 2010 [15]. Till 31 May 2022, 50% of electricity production is generated by coal combustion according to the Ministry of Power, Government of India (GOI), which is higher than that of solar and wind power [16]. As a result, coal demand has increased almost three times in the country [17], which has led to the diversion of thousands of hectares of forest land for mining operations. These surface and sub-surface mining activities led to the removal of topsoil and vegetation, the disintegration of surface and sub-surface hydrology, habitat loss, and changes in land use and land cover [18,19,20].

The densely populated and industrialized areas of northern India are one of the highly polluted regions. The sources of pollution are attributed to the increasing emissions from vehicles, industries, coal-based power plants, brick kiln industries, mining activities, forest fires, and biomass burning, [21,22,23,24,25], which affects the air, soil, and water quality. In the northern parts of India, the emissions from the thermal power plants account for 15% of particulate matter 2.5 (PM_2.5_), 30% of nitrogen oxides (NO_x_), and 50% of sulfur dioxide (SO_2_) emitted into the atmosphere. CO_2_ emission increased by 5.6% from Indian thermal power plants during 2001–2010 [25]. SO_2_ levels have increased by 56% since 2000 and continue to grow in the country to become the world’s second-largest emitter after China [26,27]. Similarly, nitrogen dioxide (NO_2_) has increased by 24.9% in Chennai, 6.13% in Delhi, and 16.8% in Hyderabad during 2005–2014 [28]. Furthermore, heavy metals such as zinc, iron, copper, manganese, lead, and nickel are widely detected in the water bodies in and around [29,30,31,32,33,34]. Additionally, leaching from surrounding landfills and possible toxic byproducts contaminate the underlying soil and even the groundwater [20,35,36].

Singrauli, also known as the energy hub of India, with a number of coal-based TPPs and extensive coal mining activities, is one of the highly polluted regions [20,22,37]. As a result, high concentrations of PM, NO_2_, and SO_2_ are severely affecting air quality of the region [38]. Black carbon (BC), capable of direct impact on solar radiation absorption is also found in the area with peaks during early morning and evening hours [22]. High concentrations of heavy metals such as cadmium, lead, arsenic, and nickel are reported in water and soil samples collected near TPPs in this region [20,25,32,35].

Regular monitoring of soil, water, and air quality of such highly polluted regions is very important to plan for future strategies and mitigate the adverse impacts on human health, and ecology. The quantitative and qualitative monitoring can be carried out using ground and satellite sensors which help us to understand the dynamics of pollutants and long-term impacts for forecasting future scenarios, especially with respect to climate change. However, developing countries like India have limited resources and logistic support for an extensive monitoring network. For instance, under NAMP (National Air Quality Monitoring Program), there are 804 air quality monitoring stations that provide 104 observations per year per location covering an area of 3.287 million sq km [39]. Furthermore, CAAQM (Continuous Ambient Air Quality Monitoring Program) has 303 continuous air quality monitoring stations in India (cpcb.nic.in). Although, in practice, the number of sampling sites depends on population, city size, terrain, spatial variation of pollutant concentration, and resource availability. The in situ observations are accurate but in India, the density of such monitoring network is low and are unevenly distributed [40]. On the other hand, satellite data provides an extensive temporal and spatial coverage of various features that could be used to extract relevant information for detailed environmental analysis [41,42]. It can also provide information on pollutant trajectories and pathways from local to global scales [24,43]. 

There are certain gaps in monitoring and analysis of pollutants, such as (a) many studies focused largely on particulate matter [22,44], or are focused on limited pollutants [26,43]; (b) most studies have monitored pollution for a shorter time period between 1–3 years [22,44], however, long-term (≥15 years) analysis of air pollutants in India are difficult to be carried out due to various reasons; (c) geo-accumulation index of the soil in the vicinity of coal mining areas and thermal power plants (TPPs) is rarely carried out.

In view of the increasing pollution, understanding of long-term annual/seasonal variations and forecast of concentrations of different pollutants (CO_2_, NO_x_, SO_2,_ PM, and CH_4_), the long-term trend and variability of pollutants provide important information to policymakers to find ways and means to control the emissions. The main objectives of the present study are to (a) analyze the long-term trend (15 years) and seasonal variations of pollutants, CO_2_, NO_2_, SO_2_, and CH_4_, with respect to TPPs in the Singrauli region using satellite data; (b) predict the concentration of these pollutants in the study area for the next 5 years, and (c) evaluate the geo-accumulation index for heavy metals using field observation of soil contamination in the study area. 

## 2. Materials and Methods

### 2.1. Study Area

**Figure 1:**
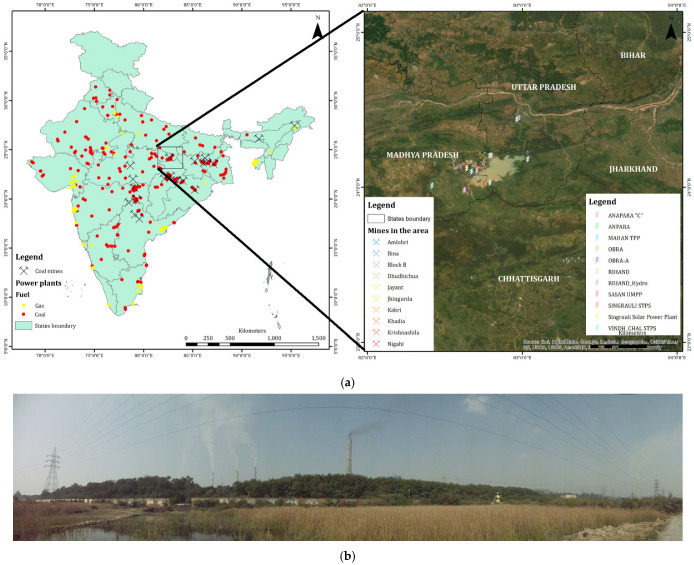
(**a**) Location of power plants (Gas and Coal) and coal mines in India (**Left**), Map of study area, Singrauli, with the location of thermal power plants and coal mines (**Right**). (**b**) Photo from Singrauli area where a number of coal-based power plants (see Table 1) and coal mines are located; all these activities impact the atmosphere and air quality in the areas (photo taken by Ramesh Singh).

There are many coal-based, gas-based thermal power plants (TPPs) in India as shown in Figure 1a, out of which the Ministry of Environment and Forest (MoEF) has identified Singrauli as a “Critically Polluted Area”. This study is carried out in Singrauli region also known as Urjanchal, “Energy Capital of India”, which is situated on the border of Uttar Pradesh and Madhya Pradesh in Central India. Singrauli region is located around 24.1960° N and 82.6676° E at an elevation 463 m, covering an area of 5675 sq km, with a population of around 1.2 million. Geologically, sedimentary rock formations belonging to the Vindhyan and Gondwana Supergroups; volcano-sedimentary rock formations of Precambrian Mahakoshal Group, and Precambrian Chhotanagpur Granite Gneiss Complex (CGGC) are present in the area. Additionally, CGGC, represented by Dudhi group of rocks, mainly including migmatitic granitic gneisses and porphyritic granite, besides the numerous metasedimentary enclaves which are present in the area [36].

In the past, the area was covered with an inhabitable dense forest with abundant natural and mineral resources. During the 1800s to 1950s, the original inhabitants and tribal communities were dependent on agricultural activities [45]. Pre-industrialization studies show that the area was covered with forest (43.35%), cropland (38.56%), and culturable wasteland (17.44%) [46]. Industrialization started with the construction of Rihand dam creating the Govind Ballabh Pant Sagar reservoir in the late 1950s. It was mainly built for irrigation purposes and hydro-power generation of 400 MW capacity. However, diverse electricity-requiring industries were established in the region instantaneously. For example, the Hindalco aluminum industry was established in 1962 followed by Kanoria Chemicals in 1964, and UP state cement corporation in 1970 [47]. During the early 1970s, the largest coal deposit was found which is being mined currently in the Singrauli region. 

We have considered an area of a 2 × 2° box (23 N, 81 E, 25 N, 83 E) in the Singrauli region (Figure 1a) for carrying out our analysis. There are many Thermal Power Plants (TPPs) in this region generating around 21 GW of electricity (Figure 1; Table 1) (https://endcoal.org/tracker/ accessed on 10 May 2022) which act as a stationary pollution source (Figure 1b). Details of TPPs installed in the Singrauli region since 2005 are given in Table 1. Numerous studies from the early 1990s have reported contamination in soil, water, and air in this region due to industrial development [48,49]. The climate of the study area can be characterized by hot summer and cold winter. The temperature ranges from a minimum of 2 °C during the month of January to a maximum of 47 °C during June. The average annual rainfall in the region is around 1133 mm, about 80% of which occurs during the south-west monsoon from June to September. 

### 2.2. Materials

We have carried out an air pollution analysis and soil quality index for heavy metals. For air pollution analysis, we have selected SO_2_, NO_2_, CO_2,_ and methane (CH_4_) pollutants. The daily data for SO_2_ and NO_2_ is taken from ozone monitoring and instrument (OMI) [50,51] between 2005 and 2020 with a spatial resolution of 0.25 × 0.25°. Similarly, for CO_2_, monthly data is taken from Atmospheric Infrared Sounder (AIRS) [52] between 2003 and 2016 with a spatial resolution of 2 × 2.5°. In the case of CH_4_, daily data is taken from AIRS [53] between 2003 and 2020 with a spatial resolution of 1 × 1° (Table 2). 

The soil samples were collected around coal mines and TPPs in the Singrauli region in polyethylene collection bags from a depth of 15–30 cm. The soil samples were taken from this depth to remove the contamination from the top surface due to anthropogenic activities. These samples were analyzed for arsenic, fluoride, iron, copper, chromium, manganese, zinc, and titanium [25].

### 2.3. Methods

#### 2.3.1. Air Quality Analysis

We have carried out the descriptive analysis and evaluated the minimum, maximum, mean, standard deviation, and variance of pollutants to attribute distribution of data, range of data, outliers, and errors. After the descriptive analysis, with the help of a box and whiskers plot, outliers are identified using the following conditions [54]:

*Outliers* < *Q*_1_ − (*IQR* × *1.5*) (1)

*Outliers* > *Q*_3_ + (*IQR* × *1.5*) (2)

where IQR = Inter-quartile Range, Q_1_ = First Quartile of the data, Q_3_ = Third Quartile of the data

**Figure 2:**
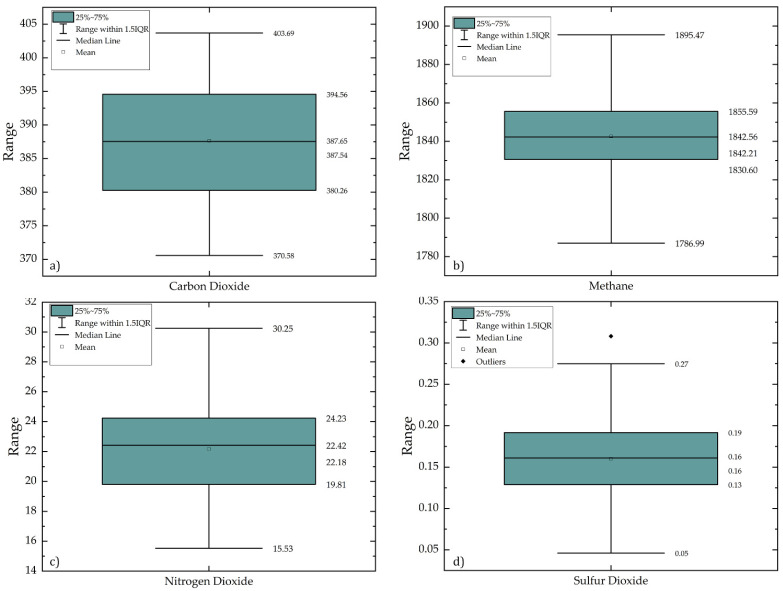
Box and whiskers plot after pre-processing (outlier and noise removal) for (**a**) carbon dioxide, (**b**) Methane, (**c**) Nitrogen dioxide, and (**d**) Sulphur dioxide.

These outliers are selected and removed from the data. As the IQR of the data will change, the box plot is again plotted and most of the outliers were removed except a few (Figure 2). The median value for CO_2_ and CH_4_ is lower than their respective mean values whereas, the median value is higher than the mean value for NO_2_ and same for SO_2_

**Figure 3:**
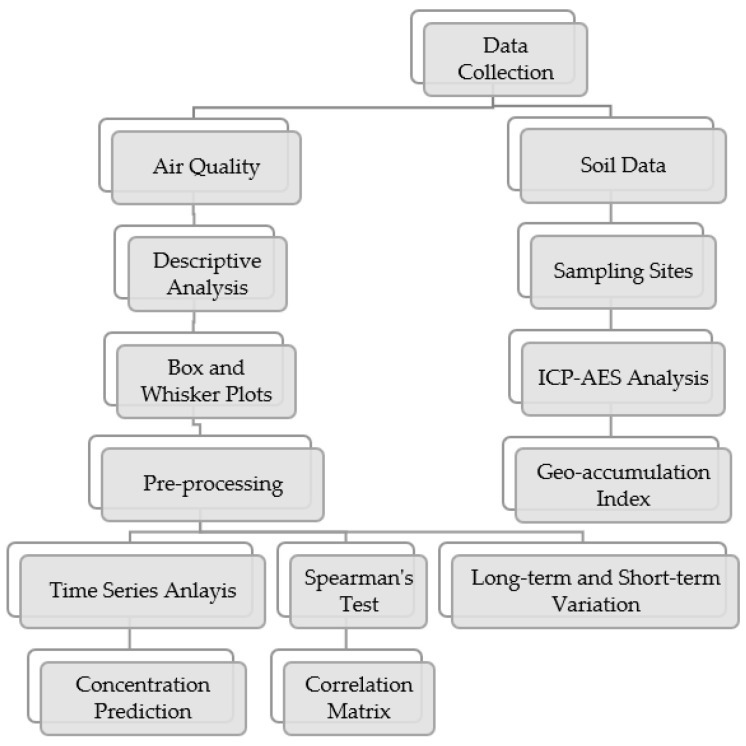
Detailed flowchart of methodology followed.

This pre-processed data is further used for time series, linear regression analysis, and henceforth for forecasting pollutant concentration. Figure 3 shows a detailed flowchart showing the methodology and analysis steps. We have used the classical multiplicative model for time series analysis using the following equation [55].

*Y*_*t*_ = *S*_*t*_ × *I*_*t*_ × *T*_*t*_ (3)

where Y_t_ = original data/predicted data, S_t_ = Seasonal component, I_t_ = Irregularity component, T_t_ = Trend component.

Seasonal, trend, and irregularity components are required to predict the pollutant concentration as expressed in Equation (3). Seasonality (*S_t_*) and irregularity (*I_t_*) components are extracted by smoothening the data using a 12-month moving average and calculating the center moving average as the seasonal data is even numbered. The mathematical equation used to calculate *S_t_ I_t_* is [55]:

(4) $$S_{t}\;I_{t}=\frac{Y_{t}\;}{CMA}$$

where Y_t_ = Original Data/Predicted Data, S_t_ = Seasonal Component, I_t_ = Irregularity Component, CMA = Centre Moving Average.

Next, the irregularity component is removed to obtain the seasonal component that is used to deseasonalize the data. It is conducted by averaging the seasonality and irregularity of individual months for the entire data set. The result thus obtained is a 12-month cyclic trend. Using this seasonal component, the data is deseasonalized using the following equation [55].

(5) $$De-seasonalize=\frac{Y_{t}\;}{S_{t}\;}$$

where Y_t_ = Original Data, S_t_ = Seasonal Component.

This deseasonalized data is used in linear regression to extract trend components using a simple linear equation [55].

*Y* = *mx* + *c* (6)

where m is the slope and c is the intercept that is obtained from linear regression and x is the time component.

Further, the trend and seasonal components are used in Equation (3) to forecast pollutants until 2025. While obtaining the trend component, three iterations with different training periods are used. The first iteration uses an initial 10 years of data for training and the next 5 years of data is used to test the success of regression followed by a forecast for the next 5 years. In the second iteration, first, 12 years of data is used for training, the next 3 years for testing, and then forecasted for 5 years. In the third iteration, first, 8 years of data is used for training and after this, the next 7 years of data is used for testing, and further forecasted for 5 years. Root mean square error and r^2^ were calculated to attribute the best-suited iteration for the forecast. Additionally, Spearman rank correlation was performed to understand the statistical significance between pollutants under observation to obtain the correlation matrix among the pollutants. It is the measure of the strength of a monotonic relationship between paired data, mathematically described as [56]:

(7) $$\rho =1-\frac{6\sum \;{d_{i}}^{2}}{n\left(n^{2}-1\right)}\;$$

where ρ = Spearman rank correlation, d_i_ = the difference between the ranks of corresponding variables, n = number of observations.

#### 2.3.2. Soil Quality Analysis

The soil samples were collected and analyzed for arsenic, fluoride, titanium, iron, chromium, lead, copper, zinc, and manganese using the inductively coupled plasma-atomic emission spectrometry (ICP-AES) at Anacon Laboratories, Nagpur, recognized by the Ministry of Environment & Forests (MoEF) which are given in Table 3 [25]. This data of heavy metal concentration in soil samples is used to estimate the geo-accumulation index.

The geo-accumulation index of heavy metals in soil samples is evaluated using the following equation as described by Muller [57]:

I_geo_ = log_2_ (Cn/1.5Bn) (8)

where Cn is the measured concentration of metal and Bn is the geochemical background values of metals as explained by Muller [57]. The background reference values are taken based on the lithology where the sites are situated. All the sites have major lithology of sandstone with minor shale so the background concentration of sandstone is used. However, Rihand dam and Hindalco TPP are situated on granitic-gneissic complex.

A factor of 1.5 is used to include a possible variation of background values due to lithogenic effects [57]. Soil quality is classified according to geo-accumulation index (I_geo_) values, i.e., unpolluted, moderately polluted, and extremely polluted (Table 4).

## 3. Results

In the Singrauli region, coal mining activities are prevalent, and the coal from these mines are used in the TPPs. The transport of coal from mining areas to coal-based power plants is the source of pollution along the roads that affect the people living in the region. The people in this region use coal for cooking and heating purposes during the winter season [22] thus elevating the levels of PM2.5 to very high. Furthermore, Indian coal has high ash content and low calorific value. Sulfur content is also less compared to coal found in the United States and China. The low calorific value and high ash content increase the emissions per kWh electricity generated. In addition, the coal mined from opencast mines such as in the study area has more ash content. Indian coal has another problem as its silica and alumina content is high, which reduces the ash collection efficiency at electrostatic precipitators (ESPs). The Indian government has mandated the use of coal after the reduction of ash content by at least 34% in critically polluted and ecologically sensitive areas. However, due to a lack of access to continuous monitoring data, compliance is uncertain [22]. We have analyzed long-term variations of air quality and have discussed long- and short-term variations of pollutants in the subsequent sections.

### 3.1. Long-Term Variations of Pollutants Associated with Thermal Power Plants

The natural and anthropogenic activities enhance CO_2_ concentrations in the atmosphere that are responsible for climate change and global warming. The CO_2_ concentrations in the atmosphere have a long-term residence time of about 300 to 1000 years. The descriptive analysis shows that monthly concentration of CO_2_ ranges from 370.58 ppm to 403.69 ppm between 2003–2016. The average CO_2_ concentration is 387.65 ppm with a standard deviation of ±8.70 and a variance of 75.52 ppm (Table 5). This shows that the minimum and maximum concentrations have a low deviation from the average concentration. It is observed that CH_4_ Concentrations ranges from 1786.99 ppbv to 1895.47 ppbv between 2003–2020 (Table 5). The average concentration of CH_4_ is 1842.56 ppbv with a standard deviation of ±20.18 ppbv and a variance of 407.14 ppbv. 

In the case of SO_2_, concentration ranges from 0.05 DU to 0.27 DU between 2005–2020, the average value is 0.16 with a standard deviation of ±0.05 DU and a variance of 0.002 DU. The NO_2_ concentration is increased from 15.53 ppm to 30.25 ppm between 2005–2020. The average concentration of NO_2_ is 22.18 ppm with a standard deviation of ±3.15 ppm and a variance of 9.92 ppm.

With the increasing energy requirement, the number of TPPs in the study area has increased since 2005 (Table 1). The power generation capacity in the study area is increased by 1000 MW in 2005. In the following years 2006 and 2007, the cumulative capacity increased to 2000 MW. Further, it increased to 4800 MW in 2012, 9920 MW in 2014, and 13,580 MW in 2017. Hence, between 2005–2017, the total power generation capacity installed in the study area is around 13,580 MW, which is almost double, as compared to capacity installed before 2005, i.e., 7584 MW. Thus overall, around 21 GW power generation capacity is installed in the study area. The increase in the number of TPPs, as well as the increase in the installed capacity of existing TPPs, has led to an increasing in the concentration of pollutants in the study area.

The long-term variation of each pollutant is plotted on an annual basis to see the effect of installation and expansion of TPPs in relation to pollutant concentrations. As the data range for each pollutant is different, a min-max normalization technique is applied for comparable results. The trend in variation of each pollutant is obtained by using the slope of the trend line of normalized data. It is observed that all the pollutants are showing an increasing trend. 

**Figure 4:**
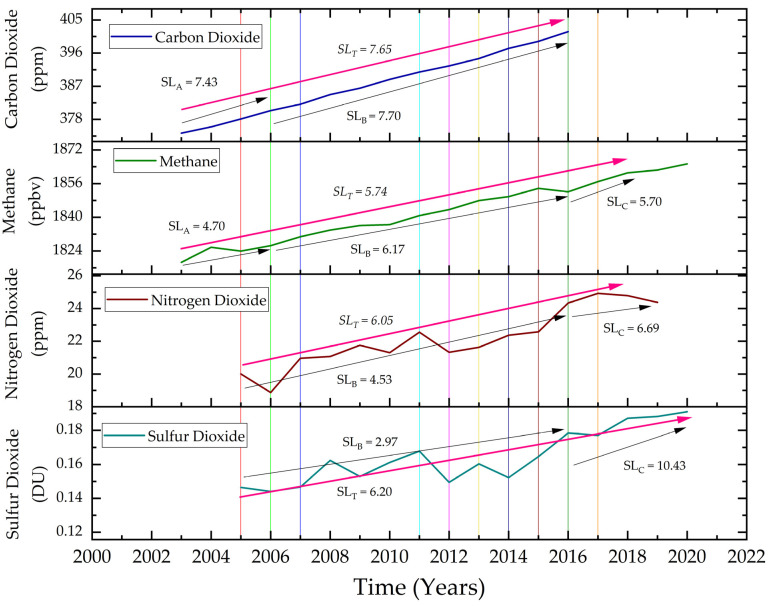
Long-term pollutant variation with respect to TPPs. The straight lines mark the installation of the new TPP. Details of new thermal power plants established are as follows: 2 in 2005, 1 TPP in 2006 and 2007, 4 in 2012, 4 in 2013, 4 in 2014, 3 in 2015, 2 in 2016 and 1 in 2017.

For the whole time period, CO_2_ has increased from 374.27 ppm in 2003 to 401.80 ppm in 2016 with a slope of 7.65 (Figure 4). Similarly, SO_2_ increased from 0.15 DU in 2005 to 0.20 DU in 2020 with a slope of 6.20 and NO_2_ increased from 20.00 ppm in 2005 to 22.00 ppm in 2020 with a slope of 6.05. Also, CH_4_ increased from 1818.64 ppbv in 2003 to 1865.34 ppbv in 2020 with a slope of 5.74. Thus, the concentration of CO_2_ increased at the fastest rate as its slope is maximum followed by SO_2_ and NO_2_, whereas, CH_4_ increased at the least rate among all four pollutants.

Furthermore, the data is segregated for different time periods to relate the variation in pollutant concentration with the installation or expansion of TPPs. The installation or expansion of TPPs are not at a uniform rate. Hence, the time period for pollutant concentration was chosen on the basis of increased power generation capacity. Three different time periods from 2003–2006, 2006–2015, and 2015–2018 are selected, during which the cumulative increase in power generation capacity in the study area is 1500 MW, 9920 MW, and 13,580 MW respectively. Thus, after 2015, as the cumulative power generation has increased to around 21 GW, it is anticipated that the concentration of pollutants will enhance in the future will further degrade air quality and associated impacts such as the formation of haze and fogs etc. It is attributed that in 1st time period CO_2_ concentration is increased with a slope of 7.43 and CH_4_ concentration is increased with a slope of 4.70. In the 2nd time period, CO_2_ concentration increased with a slope of 7.70, CH_4_ increased with a slope of 6.17, NO_2_ concentration increased with a slope of 4.53, and SO_2_ concentration is increased with a slope of 2.97. Lastly in the 3rd time period, NO_2_ concentration increased with a slope of 7.48, SO_2_ concentration increased with a slope of 10.42 and CH_4_ concentration increased with a slope of 5.70. Hence, CO_2_ increased at the fastest rate in 1st-time period and 2nd-time period, however, SO_2_ increased at the fastest rate in the 3rd-time period followed by NO_2_.

It can be observed that CO_2_ and CH_4_ have been on a continuous rise since 2003. On the other hand, NO_2_ and SO_2_ have some sinks in concentrations. This is because rainfall does not have an immediate, but a long-term effect on CO_2_ and CH_4_ [58]. Both the pollutants decrease with an increase in plant or forest cover in the area. In other words, they are not directly mixed with rainfall to decrease their concentration in the surrounding air. These pollutants are decreased as rainfall or monsoon increase the green cover in the area, which acts as a sink for their concentration. However, NO_2_ and SO_2_ can be mixed with rainfall and result in the formation of acid rain [59]. The dip in SO_2_ and NO_2_ in the year 2012 may be caused due to a 61% higher than average rainfall received in the study area [60]. Additionally, the data for both these pollutants is total column data, any variation in the atmosphere can cause a change in concentration. This may be the reason for higher uncertainty in the data. Similarly, in the year 2014, the heavy rainfall and winds are caused by the Hudhud cyclone [61]. The strong winds caused the dispersion of pollutants, reducing the concentration of pollutants, and also causing strong mixing of the pollutants which could be the cause of acid rains in the study area, showing a scavenging effect that result in the declining of pollutants in the atmosphere. 

### 3.2. Short-Term Variation of Pollutants Associated with the Thermal Power Plants

The short-term variation of each pollutant is plotted on a monthly basis (monthly average of pollutant concentration) to see the effect of installation and expansion of TPPs in relation to pollutant concentrations. 

**Figure 5:**
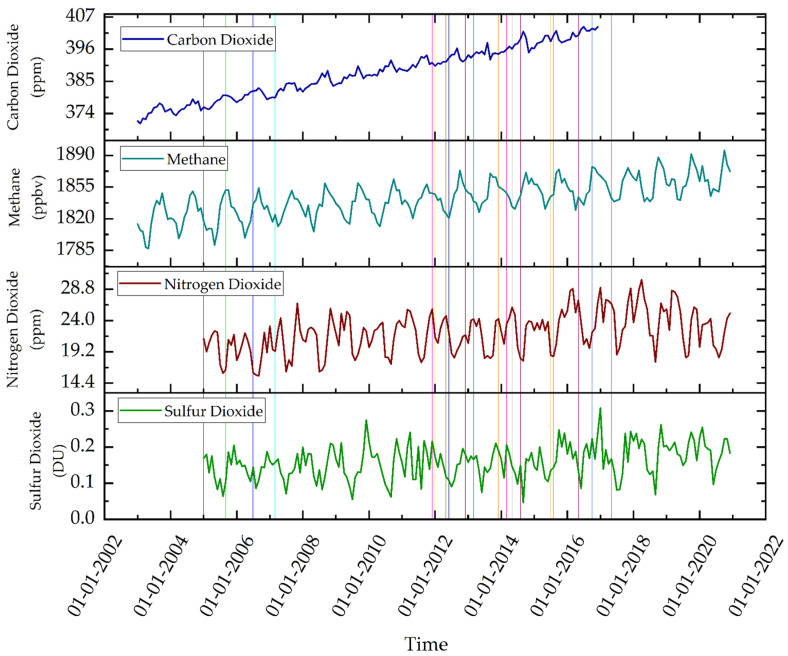
Short-term pollutant variation with respect to TPPs. The straight lines mark the installation of new TPP. Details of new thermal power plants established are as follows: 2 in 2005, 1 TPP in 2006 and 2007, 4 in 2012, 4 in 2013, 4 in 2014, 3 in 2015, 2 in 2016 and 1 in 2017.

The growing anthropogenic activities are associated with the increasing population, urbanization, biomass burning, traffic, and coal burning in TPPs and households which are the main sources of the increasing CO_2_ concentration and its various adverse impacts. During 2007, the capacity of NTPC Shakti Nagar TPP was increased in the month of March 2007 by 500 MW, increasing the cumulative capacity to 9584 MW, which resulted in a high concentration of CO_2_ in the month of June 2007. Similarly, in 2014, Reliance and Jaypee increased their capacities by 660 MW each in the month of March and May 2014, raising the total power generation to 16,844 MW, resulting in high concentrations of CO_2_ in the month of June 2014. Furthermore, Reliance TPP increased its capacity by 660 MW in the month of March 2015 and Hindalco established a new TPP with a capacity of 900 MW in the month of May 2015, thus raising the total power generation to 19,064 MW in the study area. This results in high concentrations in the month of July 2015 (Figure 5). 

CH_4_ gas emission is one of the second-highest contributors to atmospheric warming after the CO_2_, being 28 times more effective at trapping radiation and warming the planet. The net increase in CH_4_ concentration in the atmosphere is mainly due to high anthropogenic emissions such as coal mining, coal burning in TPPs, etc. The high CH_4_ concentrations can be observed in the study region. For instance, the capacity increase of NTPC Shaktinagar by 500 MW in the month of June 2012 increases the total power generation to 11,784 MW. This has resulted in the rise of CH_4_ concentration from 1820.60 ppbv in June 2012 to 1873.25 ppbv in the month of October 2012 (Figure 5). Similarly, the capacity increase of Reliance and Jaypee TPPs by 2640 MW till the month of August 2014, increasing the total power generation to 17,504 MW. This results in an increase of CH_4_ concentration from 1845.37 ppbv in August 2014 to 1859.57 ppbv in the month of September 2014. The new Hindalco TPP was established in the month of March 2015 with a capacity of 900 MW, and the expansion of NTPC Vindhyachal in August 2015 increased the total power generation capacity to 19,564 MW. This increase in power generation resulted in an increase of CH_4_ concentration from 1852.50 ppbv in March 2015 to 1870.85 ppbv in September 2015. Essar TPP increased its power generation capacity to 1200 MW in the month May 2017 thus, increasing the total power generation of 21,164 MW in the study area. This resulted in an increase of CH_4_ concentration from 1842.61 ppbv in the month of May 2017 to 1868.70 ppbv in the month of October 2017.

SO_2_ is a strong colorless gas with a pungent odor, which, upon reaction with other elements, can form harmful compounds such as Sulfuric acid and sulfate particles (PM). In such areas where huge amounts of anthropogenic emissions are observed, SO_2_ causes acid rain which is a serious threat to human, vegetation and surrounding resources. Anthropogenic activities have offset concentrations of SO_2_ entirely. For instance, Reliance and Jaypee TPP capacity increased by 660 MW each in the month of December 2013 resulting in an increase of total power generation capacity to a value of 14,864 MW. This increase resulted in an increase in SO_2_ concentration from 0.19 DU in December 2013 to 0.21 DU in the month of March 2014. In August 2014, Reliance TPP expanded its power generation capacity by 660 MW which increased the total power generation in the study area to 17,504 MW. This resulted in the sudden increase of SO_2_ concentration from 0.15 DU in August 2014 to 0.17 DU in October 2014 (Figure 5).

NO_2_ is another pungent odor gas, which can evolve in the atmosphere to form nitric acid (acid rain) and other nitrates causing a threat to human health, air quality, and the environment. It plays a major role in the production of ground-level ozone. It is also the main component of photochemical smog and particulate matter. Due to TPPs, sudden spikes are observed in the study area right after their installation. For instance, the expansion of NTPC Rihand in March 2007 by 500 MW, increased the total power generation capacity to 9584 MW. This expansion resulted in high NO_2_ concentration in April 2007. Reliance further expanded its power generation capacity in August 2014 and March 2015 by 660 MW each, resulting in an increase in total power generation capacity to a value of 17,504 MW in August 2014 and 18,164 in March 2015. This expansion resulted in sudden spikes of high NO2 concentration from 18.19 ppm to 23.21 ppm in October 2014 and from 22.65 ppm in March 2015 to 24.14 ppm in April 2015.

### 3.3. Time Series Analysis of Pollutants

**Figure 6:**
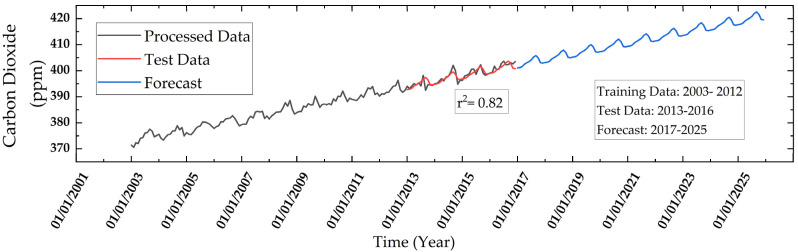
Temporal variations of monthly mean CO_2_ in the study area during 2003–2016 and forecast during 2016–2022 using linear regression.

Natural occurring CO_2_ is essential in warming the planet to make it habitable. However, anthropogenic activities have significantly increased their concentration contributing to global warming. The pre-industrial CO_2_ level was at 280 ppm and the global average crossed 400 ppm in 2018 [62], whereas the maximum CO_2_ concentration is observed to be 403.69 ppm in 2016 in the study area. The monthly CO_2_ concentrations are on an increasing trend despite its natural sinks (Figure 6) that follow a “Keeling’s Curve”. Keeling’s Curve, is a graph of CO_2_ concentration, which shows that the concentration peaks during spring and sinks during fall (autumn). 

The preprocessed data between 2003 and 2011 is used for training the regression model, whereas data from 2012–2016 is used to test the regression model. The test data between 2012–2016 is compared with predicted data and the r^2^ obtained is 0.82, thus showing a successful prediction model due to a low value of standard deviation ±8.8 with respect to a mean of 388 ppm. Using this regression model, CO_2_ concentration is predicted till 2025. The results of the regression show that the concentration of CO_2_ can increase to a maximum of 422.59 ppm and a minimum of 417.70 ppm in 2025. It shows that if no mitigation measures are taken, the CO_2_ will continue to increase and soon reach the upper limit of 430 ppm, which will overshoot the 1.5-degree global temperature rise goal, according to IPCC. 

**Figure 7:**
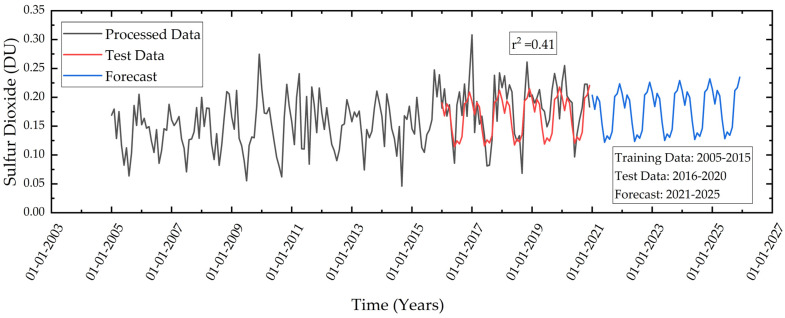
Temporal variations of monthly mean SO_2_ in the study area during 2005–2020 and forecast during 2021–2022 using linear regression.

The SO_2_ associated with anthropogenic activities play a major role in climate change [63] which is also responsible for acid rain. Secondary aerosol particles are considered to be a successor of SO_2_ and are responsible for the formation of haze. As per the natural cycle of SO_2_, high concentrations can be observed during the winter season and low concentrations during the summer season. This is related to temperature, humidity, and wind speed [64]. The pre-processed data between 2005 to 2014 is used for training the forecast model and data between 2015–2020 is used to test the model. The test data is compared with the predicted data and a low r^2^ value of 0.41 is obtained. This low r^2^ value can be attributed to the high deviation in the data i.e., the average value is almost three times the standard deviation. This shows a high uncertainty in the SO_2_ concentration data. Also, it can be observed that after 2016 data shows sudden spikes, due to which r^2^ value estimated is low (Figure 7). However, using the regression model, it is predicted that the maximum concentration can rise to 0.23 DU, and the minimum concentration can reach to 0.13 DU in 2025.

**Figure 8:**
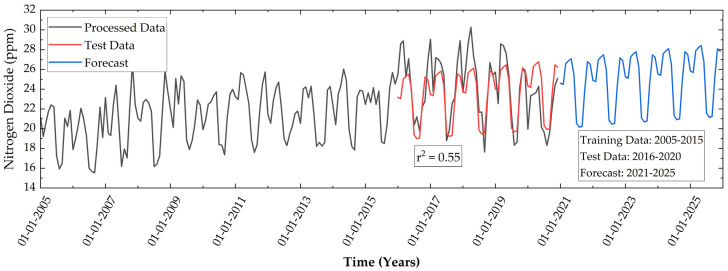
Temporal variations of monthly mean NO_2_ in the study area from 2005 to 2020 and forecast during 2021–2022 using linear regression.

Under the influence of solar radiations, which results in ground-level ozone formation, the concentration of NO_2_ during summer is low [65]. On the other hand, due to low temperature, high humidity, and low wind speed, the NO_2_ concentration increases during the winter season (Figure 8). The pre-processed data between 2005 to 2014 is used for training the forecast model and data between 2015–2020 is used to test the model. The test data is compared with the predicted data and a low r^2^ value of 0.55 is obtained. This low r^2^ value can be attributed to the high deviation in the data, i.e., the average value is almost four times that of the standard deviation. This shows a high uncertainty in the NO_2_ concentration data. However, this regression model predicts that the maximum concentration can rise to a value of 29.28 ppm, and the minimum concentration to 21.82 ppm in 2025. 

**Figure 9:**
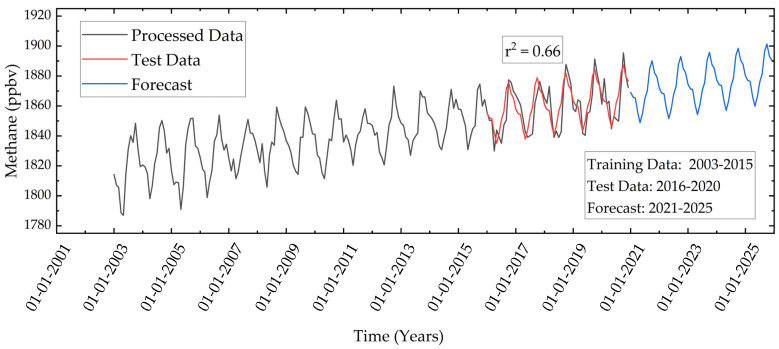
Temporal variation of monthly mean CH_4_ in the study area during 2003–2020 and forecast during 2021–2022 using linear regression.

CH_4_ is one of the short-lived climate pollutants with 28 times greater power than CO_2_ in warming the planet, nearly 60% of methane is produced by anthropogenic activities [66]. Additionally, it promotes the formation of ground-level ozone and smog. The burning of CH_4_ gas forms black carbon and volatile organic compounds [28]. Due to its catalytic nature, it is imperative to regulate its emission. An increasing trend of methane is observed in the study area (Figure 9). The pre-processed data between 2003 to 2014 is used for training the forecast model and data between 2015–2020 is used to test the model. The test data is compared with the predicted data and the r^2^ value obtained is 0.66 due to the low value of standard deviation ±19.19 with respect to the mean value of 1840 ppbv. The predicted data shows that the maximum concentration can rise to a value of 1901.35 ppbv and the minimum concentration can increase to a value of 1859.81 ppbv in 2025.

### 3.4. Spearman’s Rank Correlation

The pollutants in the environment follow a cycle, they complete their residence time and convert into other compounds through chemical reactions. For instance, CH_4_ oxidizes to CO_2_ and H_2_O, after its residence period; NO_2_ results in the formation of NO_x_ and O_3_ (ozone); NO_2_ and SO_2_ form secondary aerosols, i.e., particulate matter (PM) [67]. These aerosols are responsible for direct solar radiation absorption, which results in the warming of the planet. Additionally, they also form smog, haze, etc., which reduces visibility, thus impacting the environment. With an increase in warming, the atmospheric circulation reduces, and the accumulation of pollutants in a region increases. This leads to an increase in natural emissions of CO_2_ and CH_4_ in the atmosphere. The enhanced concentrations of these pollutants in the warming climate create a positive feedback loop. Hence, these pollutants need to be studied in relation to aerosols present in the atmosphere.

Therefore, in this section, AOD (Aerosol Optical Depth), downloaded from the NASA Giovanni portal, PM_2.5_, and PM_10_, taken from CPCB ground observations, are analyzed with other pollutants. Spearman’s rank correlation is used to correlate pollutants and aerosols in the study area. Spearman’s correlation coefficients are depicted in the lower diagonal and *p*-values are shown in the upper diagonal of the correlation matrix of Table 6. The significant correlations, at a ≥95% significance level, are shown in bold in Table 6. 

NO_2_ shows a high positive correlation with PM_2.5_ (0.60) and PM_10_ (0.66). Moreover, CO_2_ is significantly positively correlated with NO_2_ (0.47), CH_4_ (0.48), and SO_2_ (0.28). Similarly, CH_4_ has a high positive correlation of 0.59 with SO_2_, while it has a significant negative correlation of −0.23 with AOD. Hence, it shows that an increase in CO_2_ will result in an increase in NO_2_, CH_4_, and SO_2_. This is because the increase in CO_2_ changes the composition of surrounding air and results in an increase of other pollutants in the atmosphere [68]. Since NO_2_ is the precursor of secondary aerosols, NO_2_ shows a high positive correlation with PM_2.5_ and PM_10_. On the other hand, the negative correlation between CH_4_ and AOD is attributed to the short life span of CH_4_ and its early conversion to soot (BC).

### 3.5. Accumulation Index of Soil in Singrauli Region

A typical power plant uses 12,000 tons of coal per day and drains 1 Mt of waste per year. During this process, a significant number of byproducts are transferred to the surrounding environment. These byproducts have two essential routes to be released into the environment: atmospheric emission and leaching of dumped byproducts such as ash ponds, ash dumps, etc., in the surrounding soil. 

It was observed that for arsenic contamination, Obra TPP, Bina coal mines, and Khadia coal mines are categorized in class 2 of geo-accumulation index, i.e., moderately polluted. For lead, Bina coal mines’ sampling site is categorized in class 4 of geo-accumulation index, i.e., highly polluted, while, Obra TPP and Khadia coal mines are categorized in class 3 of geo-accumulation index, i.e., moderate to highly polluted. It is observed that for chromium Hindalco industries is classified in class 5, i.e., highly to extremely polluted, Rihand dam is categorized in class 4, i.e., highly polluted and Lanco TPP and Obra TPP are classified into class 3, i.e., moderately to highly polluted. In the case of zinc, the Anpara TPP site is classified in class 5 of geo-accumulation index, i.e., highly to extremely polluted, while the Bina coal mines and Vindhayachal TPP are classified in class 3 of geo-accumulation index, i.e., moderately to highly polluted.

Furthermore, we have added the geo-accumulation index for all the heavy metals to obtain the cumulative index. This shows that Hindalco industries, Anpara TPP, Bina coal mines, Khadia coal mines, and Vindhayachal TPP are the most polluted, followed by Lanco TPP, NTPC Shaktinagar, Near Vindhayanagar, and Rihand dam. On the other hand, Singrauli reservoir is least polluted as compared to other sampling sites which are moderately to extremely highly polluted. It is observed that the cumulative index is highly dependent on the variation of lead followed by zinc and chromium (Table 7).

## 4. Discussion

Singrauli region has one of the most important coal mines in the country and TPPs installed surrounding the mining area generate around 21 GW of electricity, making it an “Energy Hub”. The main air pollutants emitted by coal mining and TPPs are PM, SO_2_, NO_2_, and CO_2_ [70]. It is documented that for every kWh of coal-based electricity generation, 0.8–0.9 kg of carbon dioxide is emitted into the surrounding air [1]. The establishment of Rihand Dam which created Govind Ballabh Pant Sagar reservoir, TPPs, cement plant, and aluminum and chemical industries in the late 1950s has changed the land cover and land use pattern of the region drastically. Within a period of 1978–2010, the mining area has increased by 590%, built-up area by 350%, and cropland by 71.50%. On the other hand, open forest and dense forest has decreased by 25% and 56% [19]. Further, between 2000 and 2016, mining activities increased threefold [20]. The change in LULC such as a decrease in open and dense forests has increased the surface temperature by 6.69 °C during 2005–2015 [71]. 

Our study shows that during 2003 and 2015, many TPPs generating 9920 MW of electricity were established and expanded in the study area. During this period, CO_2_ increased from 374.27 ppm in 2003 to 399.20 ppm in 2015 and CH_4_ increased from 1818.64 ppbv in 2003 to 1853.75 ppbv in 2015. During 2005–2015, SO_2_ increased from 0.15 DU to 0.17 DU (~15% increase) and NO_2_ increased from 20.00 ppm to 22.57 ppm (~13% increase). Ground observation reported that CO_2_ increased by 57.94%, SO2 increased by 57.50%, and NO_x_ increased by 60.72% during 2001–2010 [72]. In the same time period, CO_2_ increased by 54.15%, SO2 increased by 52.44%, and NO_x_ increased by 56.40% in India [72]. Also, observations are reported where SO_2_ has increased around ~25% between 2005–2012 [43] and column NO_2_ has increased ~4% between 2005–2010 [65] in India.

During 2015–2020, our study shows that TPPs’ installation/expansion increased the electricity generation by 3660 MW, raising the total generation to 21,164 MW. During the 2015–2016 period, CO_2_ increased from 399.65 ppm to 401.80 ppm. During 2015–2020, CH_4_ increased from 1853.75 ppbv to 1865.33 ppbv, and SO_2_ from 0.17 DU to 0.20 DU. Similarly, it was observed that in the year 2016–2017, PM_2.5_, PM_10_, Total Suspended Particles (TSP), NO_2,_ and SO_2_ concentrations present in the study area were higher than the National Ambient Air Quality Standards of India [38]. 

During the COVID-19 pandemic (2019–2020), the lockdown was imposed in many countries resulting in reduced air pollution levels [24]. Essential services such as electricity generation from TPPs and mining activities were also reduced during lockdown [73]. However, during this time, the air quality of Singrauli did not improve much, whereas other locations such as Delhi, Mumbai, etc. experienced reduced air pollution levels [74]. The same can be observed in our study, where, SO_2_ has increased from 0.19 ppm in 2019 to 0.20 DU in 2020 and CH_4_ has increased from 1862.44 ppbv in 2019 to 1865.37 ppbv in 2020.

In a work carried out by Guttikunda and Jawahar [75], it was forecasted that by 2030, the power generation capacity of the study area will increase by 170% which will result in a coal consumption increase by 170%. This project showed an increase in SO_2_, NO_2_, and CO_2_ by 169.2%, 132.38%, and 169.10%, respectively. In our study, on the basis of past trends, we predicted pollutant concentration in the study area from 2021–2025. This forecast shows that CO_2_ increases from 374.27 ppm in 2003 to 422.59 ppm in 2025, SO_2_ increases from 0.15 DU in 2005 to 0.23 DU, NO_2_ increases from 20.00 ppm to 29.28 ppm, and CH_4_ increases from 1818.64 ppbv to 1901.35 ppbv.

The soil analysis of our study conducted on samples collected in the year 2015 [25] shows heavy metal contamination in and around TPPs and mining areas. We found that the soil samples of the region are highly to extremely polluted in the case of Cr, Pb, and As, and unpolluted to moderately polluted for Mn, Ti, and Fe. Similarly, Agrawal et al. [35] conducted a quantitative estimation of heavy metals in the soil around TPPs from March 2005 to February 2008. The 256 samples collected during pre-monsoon and post-monsoon reported a high concentration of heavy metals in the area. The average maximum concentration of cadmium, lead, arsenic, and nickel in soil was observed to be 0.69, 13.69, 17.76, and 3.51 mg/kg, respectively [35]. 

### Limitations and Recommendation

The previous studies conducted in the Singrauli region are either short-termed or are focused on fewer pollutants. Our long-term study analyzes the variation of pollutants with respect to TPPs and forecasts their concentration on the basis of past trends using satellite-derived data. However, the limitations of this study are twofold. Firstly, while the satellite data provide broad spatial and temporal coverage, the ground-observation data are more accurate. Additionally, satellite data obtained is difficult to validate in case of a lack of ground monitoring stations. So, for high accuracy, a dense network of ground monitoring stations is required. However, in developing countries such as India, dense monitoring grid is not economical. Secondly, the satellite data available for CO_2_ has a spatial resolution of 2 × 2.5°, for CH_4_, the spatial resolution is 1 × 1°. This means that a single grid will cover a very large area of the order of thousands of sq. km. This might not be accurate for point location studies. Thus, the concentration of the pollutants may not represent a very distinct and clear picture. Hence, better resolution satellite data will provide a more comprehensive and rigorous analysis of the concentration of pollutants.

The observed trend and forecast of our study show a continuous rise in pollutant concentration in the region. Their long-term exposure can cause an adverse impact on the environment and various health impairments in humans. For instance, if more and more pollutants are loaded into the atmosphere, the global temperature will continue to rise. This can cause frequent extreme events, the rise of sea level, drought, change in rainfall patterns, etc. Our study shows that if no mitigation measures are taken, it will be difficult for India to fulfill the Paris agreement goals to curb emissions by 2030. It is also articulated that CO_2_ concentration has already reached 400 ppm, which is the limit defined by IPCC to control global temperature rise below 1.5° and it can rise to 422 ppm in 2025. Hence, it is high time to take mitigation measures and control pollution levels at the regional and national levels. For instance, the short-lived pollutant CH_4_, if controlled, can show a near-term curb in temperature rise, thus helping in achieving Paris Agreement goals. Additionally, at present, non-renewable energy sources account for more than 50% of electricity production. The shift toward renewable sources such as solar, wind, and hydropower will reduce greenhouse gases in the environment. Zero waste approaches in TPPs and efficient production equipment for minimum emissions need to be implemented. Last, strict emission rules for industries and TPPs will help reduce the pollution and, by extension, climate change and deaths caused by ambient air pollution.

## 5. Conclusions

Singrauli is one of the most highly polluted regions, owing to high emissions from TPPs, coal mining, and numerous industries. In this study, the long-term and short-term variations of air pollutants with respect to TPPs in the region are analyzed using satellite data. This study shows that annual average concentration of CO_2_ has increased from 374.27 ppm in 2003 to 401.80 ppm in 2016, SO_2_ concentrations have increased from 0.15 DU in 2005 to 0.20 DU in 2020, NO_2_ concentrations have increased from 20.00 ppm to 22.00 ppm in 2020, and CH_4_ concentration has increased from 1818.64 ppbv in 2003 to 1865.34 ppbv in 2020. The long and short-term variation concludes that pollutants’ concentration suddenly increased in 2007, 2014, 2015, and 2017, which coincided with the installation or expansion of TPPs in the study area. This concludes that increased TPPs and coal mining in the area are increasing the pollutant concentration in the atmosphere. The forecast of the study states that the concentration of CO_2_, NO_2_, SO_2_, and CH_4_ in the year 2025 will rise to 422.59 ppm, 29.28 ppm, 0.23 DU, and 1901.35 ppbv, respectively, in the Singrauli region. It was observed that CO_2_ is significantly correlated to all other pollutants using Spearman’s rank correlation test, while CH_4_ and SO_2_ have a strong correlation with each other. Additionally, NO_2_ is significantly related to PM_2.5_ and PM_10_. Furthermore, this study concludes that the soil of the Hindalco industries, Anpara TPP, Bina coal mines, Khadia coal mines, and Vindhayachal TPP are the most highly polluted with heavy metals, while Singrauli reservoir is the least polluted. Thus, the air and soil of the Singrauli region is highly polluted. After spending some time in the area during the field visit, we are really concerned about how so many people live in such a highly polluted region. There are no records of patients suffering from different kinds of diseases, but early morning and in the evening, there is a huge lineup of patients seen at the health clinic and in the hospitals. The present study will attract the attention of Government to take steps to save the lives of people living in the surrounding areas by taking proper mitigation measures to alter the course of the pollution.

## Figures and Tables

**Table 1:** Location of various TPPs in the study area showing installation date and further expansion months. These details are extracted from the individual website of the corporations that have installed coal-based TPPs in the study area.

S. No.	Name of TPP	Location	Installation/Expansion Month	Capacity (MW)
1	NTPC	Rihand	January,2005	500
2	NTPC	Rihand	September, 2005	500
3	NTPC	Vindhyachal	July, 2006	500
4	NTPC	Vindhyachal	March, 2007	500
5	Lanco	Anpara	December, 2011	600
6	Lanco	Anpara	January, 2012	600
7	NTPC	Rihand	May, 2012	500
8	NTPC	Vindhyachal	June, 2012	500
9	NTPC	Vindhyachal	March, 2013	500
10	Essar	Sasan	December, 2012	600
11	Reliance	Sasan	March, 2013	660
12	Reliance	Sasan	December, 2013	660
13	Jaypee	Nigre	December, 2013	660
14	Reliance	Sasan	March, 2014	660
15	Jaypee	Nigre	March, 2014	660
16	Reliance	Sasan	May, 2014	660
17	Reliance	Sasan	August, 2014	660
18	Reliance	Sasan	March, 2015	660
19	Hindalco	Baragawon	July, 2015	900
20	NTPC	Vindhyachal	August, 2015	500
21	UPRVUNL	Anpara	May, 2016	500
22	UPRVUNL	Anpara	October, 2016	500
23	Essar	Sasan	May, 2017	600

**Table 2:** Details of the sensor mounted on the satellite and the spatial resolution of data collected.

S. No.	Pollutant	Unit	Sensor	Data Time	Spatial Resolution
1	SO_2_	DU	OMI	2005–2020	0.25 × 0.25°
2	NO_2_	ppm	OMI	2005–2020	0.25 × 0.25°
3	CO_2_	ppm	AIRS	2003–2016	2 × 2.5°
4	CH_4_	ppbv	AIRS	2003–2020	1 × 1°

**Table 3:** Soil sampling location in the study area and concentration of arsenic, fluoride, titanium, chromium, copper, lead, zinc, and manganese.

S.No.	Sampling Site	Latitude	Longitude	As	F	Ti	Fe	Cr	Pb	Cu	Zn	Mn
	Acceptable Limit (WHO, 1996)			2 mg/kg	48 mg/kg	300 mg/kg	48 mg/kg	42 mg/kg	2 mg/kg	30 mg/kg	60 mg/kg	450 mg/kg
1	Kakri Coal Mines	24°10′25″	82°45′32″	1.5	3.6	1540.5	9502.9	29.0	12.5	10.3	46.4	253.8
2	Near Bina Coal Mines	24°9′55″	82°44′46″	1.0	4.87	739.1	11,518.9	23.4	14.6	4.1	49.2	142.8
3	Bina Coal Mines	24°9′25″	82°4′40″	2.0	5.32	2254.7	8251.5	141.2	35.1	37.1	119.2	632.9
4	Rihand Dam	24°12′30″	83°00′05″	2.5	2.84	2794.3	22,424.7	65.0	5.1	12.2	31.8	1187.8
5	NTPC Shaktinagar TPP	24°5′55″	82°42′33″	1.3	6.1	6276.8	11,965.0	89.1	3.4	40.2	67.5	380.5
6	Vindhayachal TPP	24°5′18″	82°40′55″	2.6	3.9	2275.0	20,126.1	94.8	31.8	14.2	120.1	485.2
7	Lanco TPP	24°12′22″	82°48′44″	1.0	1.4	2553.3	43,610.6	211.1	7.0	3.3	79.4	585.9
8	Anpara TPP	24°11′27″	82°47′51″	0.9	0.83	6878.2	43,129.7	93.3	13.1	29.6	492.2	701.3
9	Hindalco Industries	24°13′05″	83°02′06″	1.3	2.03	3774.6	44,302.1	107.2	70.1	16.4	108.7	735.8
10	Obra TPP	24°26′36″	82°59′05″	4.1	3.8	2031.3	30,121.4	95.8	70.4	17.3	70.9	521.3
11	Near Vindhayanagar	24°04′53″	82°39′15″	2.1	0.33	2478.6	15,511.9	33.0	17.6	7.9	39.5	354.7
12	Between Bina and Kakri	24°09′37″	82°45′39″	1.8	5.81	1510.5	13,463.3	25.3	11.8	10.2	30.8	286
13	Bina Coal Mines	24°09′6.8″	82°46′01″	3.6	16.9	2322.0	28,976.0	99.3	84.6	24.2	64.4	558.2
14	Khadia Coal Mines	24°06′54″	82°43′26″	3.5	28.5	1737.6	29,921.4	93.9	80.9	23.7	65.3	484.2
15	Renusagar TPP	24°10′37″	82°47′26″	2.6	43.5	1871.5	15,249.8	30.4	9.7	4.9	83.9	359.9
16	Singrauli Reservoir	24°07′58″	82°48′02″	1.5	37.8	522.4	7261.3	13.7	7.5	2.0	32.1	221.3

**Table 4:** Division of class for Geo-accumulation index according to Muller, 1979 [57].

Class	Values of *I_geo_*	Soil Quality
0	*I* ≤ 0	unpolluted
1	0–1	unpolluted to moderately polluted
2	1–2	moderately polluted
3	2–3	moderately to highly polluted
4	3–4	highly polluted
5	4–5	highly to extremely high polluted
6	*I* ≥ 5	extremely high polluted

**Table 5:** Long-term statistical summary of pollutant concentrations in the study area.

S.NO	Parameter	Acceptable Limit	Minimum	Maximum	Average	Standard Deviation	Variance
1	Carbon dioxide (ppm)	400 ppm	370.58	403.69	387.65	±8.70	75.52
2	Methane (ppbv)		1786.99	1895.47	1842.56	±20.18	407.14
3	Nitrogen dioxide (ppm)	0.053 ppm	15.53	30.25	22.18	±3.15	9.92
4	Sulfur dioxide (DU)	0.10 DU	0.05	0.27	0.16	±0.05	0.002

**Table 6:** Correlation matrix of trace gases. The lower diagonal values are the correlation coefficient, and the upper diagonal are the *p*-values, signifying the correlation intensity among pollutants.

	AOD	SO_2_	NO_2_	Methane	PM_2.5_	PM_10_	CO_2_
AOD	1.00	0.76	0.86	0.45	0.44	0.96	0.03
SO_2_	0.09	1.00	0.76	0.03	0.19	0.22	0.00
NO_2_	0.55	0.09	1.00	0.76	0.03	0.01	0.00
CH_4_	**−0.23**	**0.59**	0.09	1.00	0.15	0.10	0.00
PM_2.5_	−0.24	0.39	**0.60**	0.42	1.00	0.00	NA
PM_10_	0.02	0.36	**0.67**	0.48	0.82	1.00	NA
CO_2_	**0.20**	**0.28**	**0.47**	**0.48**	NA	NA	1.00

Bold: Significant correlations at a ≥95% significance level.

**Table 7:** Accumulation index calculated using Muller formula and divided into classes as described in Table 7 in Singrauli region. The background values used are As (1.5), Ti (3400), Fe (14,200), Cr (4.1), Pb (19), Cu (10), Zn (39), and Mn (390) for Rihand dam and Hindalco TPP. For all the other sites, background values taken are As (1), Ti (1500), Fe (9800), Cr (35), Pb (7), Cu (4), Zn (16), and Mn (850) [69].

Location	As	Ti	Fe	Cr	Pb	Zn	Cu	Mn	Cumulative Index
Kakri Coal Mines	1	0	0	0	1	1	1	0	4
Near Bina Coal Mines	0	0	0	0	1	2	0	0	3
Bina Coal Mines	1	1	0	2	2	3	3	0	12
Rihand Dam	1	0	1	4	0	0	1	0	7
NTPC Shaktinagar TPP	0	2	0	1	0	2	3	0	8
Vindhayachal TPP	1	1	1	1	2	3	2	0	11
Lanco TPP	0	1	2	3	0	2	0	0	8
Anpara TPP	0	2	2	1	1	5	3	1	15
Hindalco Industries	0	0	2	5	2	1	2	1	13
Obra TPP	2	0	2	3	3	2	2	0	14
Near Vindhayanagar	1	1	1	1	1	1	1	0	7
Between Bina and Kakri	1	0	0	0	1	1	1	0	4
Bina Coal Mines	2	1	1	1	4	2	3	0	14
Khadia Coal Mines	2	0	2	1	3	2	2	0	12
Renusagar TPP	1	0	1	0	0	2	0	0	4
Singrauli Reservoir	0	0	0	0	0	1	0	0	1

## Data Availability

The data used in this work is freely available from the sources as mentioned in the acknowledgements.
